# Abortion in Domesticated Animals

**Published:** 1897-03

**Authors:** Charles F. Dawson

**Affiliations:** Veterinary Inspector, Bureau of Animal Industry


(To be continued.)

---

### ABORTION IN DOMESTICATED ANIMALS.

By Charles F. Dawson,
VETERINARY INSPECTOR, BUREAU OF ANIMAL INDUSTRY.

(Continued from page 79.)

It will be seen from the foregoing remarks that abortion in our animals may be caused by an almost infinite number of conditions. In the case of the human family, with the possible exception of frequent bodily injuries, women are subject to and do abort from many of the same causes which are said to produce abortion in the lower animals. We find in all the standard works on midwifery, and it is a common experience of many persons, that abortion may be caused by numerous agencies, the most frequent being uterine hyperæmia and inflammations, over-frequent coitus, fevers, noxious gases in the atmosphere, states of the nervous system produced by fright, anxiety, sudden losses, shock, constant suckling in those who become pregnant during lactation, obstipation, excessive vomiting of pregnancy, alcoholism, trifacial neuralgia, albuminuria, abnormalities of the pelvis, inflammatory adhesions preventing the normal development of the gravid uterus, an atonic condition of the uterus, malnutrition, severe diarrhœa, tumors, etc. Knowing that any one of these may be operative in producing abortion, is it not reasonable to suppose that some of the same causes may also be operative in the lower animals?

In support of the theory held by many persons, both lay and professional, that strong mental impressions upon the mother are reflected upon the fœtus in utero, I will mention two cases where circumstances seem to narrow the question down to a point where we could hardly account for the conditions present on any other theory. Dr. Theobald Smith[1] had occasion to investigate tuberculosis in a herd of cattle, and used as a diagnostic agent hypodermatic injections of tuberculin. The injections were made in the shoulder and neck regions. In Case No. I. he found a twin-pregnancy, each fœtus being about sixty millimetres long. In each of these fœtuses he found a blood-red spot situated anterior to the shoulder-joint and surrounded by a tributary zone of injected blood-vessels which extended in fœtus No. 1 nearly to the occiput, down the sides of the neck to the median ventral line, and also down the lateral aspect of the forelimb to the carpus. In fœtus No. 2 the left side was more affected than in fœtus No. 1. The hemorrhagic area extended from near the median dorsal line ventrad to the shoulder-joint, a distance of about six millimetres. This vascular injection extended not only over the area described in fœtus No. 1, but also nearly encircled the eye, and extended beyond the ear to the top of the head.

In Case No. II. the fœtus was seventy millimetres long. In front of the left shoulder there was a blood-red spot, suggesting a cutaneous hemorrhage. There was no anastomosing network of bloodvessels as in the fœtus in Case No. I.

In Case No. I. several injections of tuberculin were made, but no definite note was made of the exact points at which they were made. It is highly probable that both sides of the neck were injected, for in Case No. II. careful note was made of the side and exact location of the point of operation, and the post-mortem examination revealed the fact that the hemorrhagic area in the fœtus exactly corresponded with the seat of the tuberculin-injection in the mother.

Dr. Smith examined a number of fœtuses of more advanced age and several of the same age, but did not find similar lesions; and while he does not wish to put forth his discovery as a hypothesis to explain the relationship existing between the maternal organism and that of the fœtus in utero, it seems to me that these authenticated cases may be effectively used as an argument by those who believe that "birth-marks" are due to some disturbance of the nervous system of the mother being manifested in the fœtus.

In concluding his article, Dr. Smith says: "It is probable that

[1] The Veterinary Magazine, 1895, vol. ii. No. 2.

we must realize a definite combination of certain conditions before this remarkable effect may be actually seen. These are a certain age-limit of the fœtus, a certain time-limit between the tuberculin-injection and the autopsy, and perhaps a certain impressionable condition of the mother."

Possibly the blow upon the head given at the time of slaughter of the mother may have had something to do with producing part or all of the extravascular condition of the skin of the fœtus. Granting this, the theory of the reflection of maternal impressions is not disproved, and we are met with the knowledge that this possible cause was of short duration, and of an entirely different character from the tuberculin-injections, which were repeated and operative for a much longer time, and in an entirely different way.

In human medicine we cannot, of course, advance any convincing proof of the theory, held by many persons, that strong mental impressions may affect the fœtus in utero, and evidence themselves by causing certain abnormalities in the offspring. We find recorded in standard works on midwifery statements of authors who take up valuable space to relate the stories of the mothers of children so afflicted concerning the time and character of the circumstances which caused their children to be "marked". Many of these stories contain much strong circumstantial evidence, and would tend to show that there is a most intimate bond of union existing between the sympathetic nervous system or "mind" of the mother and the child she is developing.

If it is true that the vasomotor nervous system of the mother may be sufficiently shocked by a hypodermatic injection of tuberculin, which in a healthy non-tuberculous animal does not even cause a rise in temperature, could we not reason that the results of physical impressions upon the pregnant uterus and its contents are probably limited mainly by the intensity of the impressions received; that many of the cases of sporadic abortion are the outcome of these impressions, and that the differences between the mere transient vascular dilatations noted in the fœtus by Dr. Smith and a complete separation of the fœtal and maternal structures are those of degree only.

Having discussed the subject of sporadic abortion, and expressed my belief that the conditions mentioned are operative in causing it, I will now take up the other variety. Much has been written about infectious abortion, but we know very little regarding its etiology. It is hoped that this preliminary paper may be followed by others which will throw more light upon the subject.

In looking over the literature of the subject one is impressed with the similarity of the prescribed methods of treatment. Even those who try to explain all cases of abortion as being due to one or other of the causes already mentioned in this paper invariably prescribe a mode of treatment which would of itself indicate that, even though their views as to etiology be divergent, they can all meet upon a common ground as regards treatment.

Those who believe that odors, sympathy, fright, etc., are the factors in causing animals to abort could with perfect consistency adopt the cleansing methods of treatment, because by vaginal douches and removal of all material soiled by the discharges they get rid of the uterine *débris* which, were they not so treated, would decompose and give off disagreeable odors.

Those who believe that all cases of abortion are due to infection could, for obvious reasons, also adopt the same sanitary measures; and because each side to the controversy has had more or less success in combating the disease by the adoption of the same sanitary measures each holds to its respective views regarding the etiology.

That there is a form of abortion in animals having as its cause a contagium cannot be doubted. Opinions have been handed down by investigators favorable to the contagium theory, and numerous experiments have been recorded which, while they give no direct evidence supporting the theory, show that there is sufficient evidence for their formulators to make known their views without much fear of successful contradiction.

